# Preparation and characterization of SPE column with *smart green* molecularly imprinted polymers materials for selective determination of S-metolachlor herbicide

**DOI:** 10.1038/s41598-025-87685-2

**Published:** 2025-01-24

**Authors:** Dominika Rapacz, Katarzyna Smolińska-Kempisty, Joanna Wolska

**Affiliations:** Department of Process Engineering and Technology of Polymer and Carbon Materials, Wroclaw University of Science and Technology, Wybrzeże Wyspiańskiego 27, Wrocław, 50-370 Poland

**Keywords:** S-metolachlor, Solid phase extraction, *Green* molecularly imprinted polymers, Sorption, Desorption, Green chemistry, Environmental monitoring, Analytical chemistry, Sensors, Environmental monitoring

## Abstract

The presence of traces of herbicides in ground and surface waters can have adverse impacts on humans and the environment. Therefore, developing a highly selective and reusable adsorbent for monitoring water quality has become important. This article describes smart *green* molecularly imprinted polymers (MIPs) as selective sorbents of S-metolachlor herbicide for solid phase extraction (SPE). Combining the MIP-SPE column with HPLC chromatography provided a quick and accurate method for determining the real concentration of S-metolachlor in water samples. The sorption capacity of the MIP-SPE column was almost twice that of the non-imprinted polymer column for SPE extraction. The bed can be regenerated up to three times before it loses its original sorption properties. A method has been developed whereby 6 ml of water can achieve an average recovery rate of 98% for sorbent. During the selectivity study from multicomponent solution, the calculate imprinting factor for MIPs was calculated to be 10, while MIPs sorb S-metolachlor 10 times better than atrazine, 12 times better than fenoxaprop-P-ethyl and 33 times better than glyphosate. The pseudo-second-order kinetic model was in good agreement with the experimental values obtained.

**Supplementary Information:**

The online version contains supplementary material available at 10.1038/s41598-025-87685-2.

## Introduction

Maize, wheat, and rice are the three main cereal crops grown on Earth, providing more than 40% of the world’s caloric needs. Approximately 197 million hectares of corn are cultivated annually, and half of the world’s maize production is accounted for in the Americas^1^. Agrochemicals, including fertilizers, herbicides, pesticides, insecticides, and plant-growth hormones, are widely used in agriculture to increase production efficiency to meet the needs of a steadily growing global population^2^. S-metolachlor is one of the most persistent soil-applied herbicides belonging to the chloroacetanilide group, used for the pre-emergence control of small-seeded broad-leafed weeds and annual grasses, among others, in maize, cotton, and soybean crops^3^. It is relatively well soluble in water and is weakly sorbed to soil particles, giving it the potential to permeate into groundwater^4^.

The sustainable use of agrochemicals is important not only for boosting crop yield but also for human well-being and health, as well as the protection of water resources from farm pollution^5^. In addition to contamination from plant protection products, ground, surface, and drinking waters can contain micropollutants with heavy metals, medications and pharmaceuticals, dyes, drugs, and the endocrine disrupting system. The micropollutants present in water often occur at deficient concentrations, from ng × L^−1^ to µg × L^−1^, which means that many traditional analytical methods are not sensitive enough to detect and remove these contaminants^6^. Widespread analytical tools used to determine micropollutants are liquid-chromatography coupled to mass spectrometry (LC-MS), gas chromatography linked to mass spectrometry (GC-MS)^7^, and capillary electrophoresis^8^. The detection of S-metolachlor using molecularly imprinted polymers in samples containing micropollutants is typically conducted through the use of HPLC or GC. The precise parameters associated with this methodology are further delineated in the review^9^.

Enrichment and pre-concentration methods used in preparing samples for analysis are crucial in determining the trace concentration, especially for complex environmental samples, regardless of the type of contaminant or sample matrix. Solid phase extraction is an extensively used clean-up, isolation, and concentration technique because of its ease of use, cost, and low solvent consumption^10^. The types of SPE sorbents include, among others, normal, reversed, and ionic phases, however, unsatisfactory selectivity caused by co-elutes with interfering compounds excludes their use in effective and reliable analyses. Molecularly imprinted polymers (MIPs) have proven to be a good selective SPE substrate due to their specific ability to recognize the target analyte molecule. Moreover, MIPs are stable, relatively inexpensive, easy to synthesize, and have potential affinity for a wide range of interest analyte molecules. The application of *green chemistry* principles has become increasingly popular in the field of molecular imprinting to improve the performance of MIPs by reducing their negative impacts. The design of *green* MIPs poses a significant challenge to researchers, primarily due to the lack of solubility of the majority of monomers in water^9,11^. The greenification list of principles encompasses the selective implementation of environmentally friendly materials and reagents in waste disposal^11,12^.

Smart polymers are a class of functional materials that are able to respond to environmental stimuli such as temperature, pH, or light. Poly(N-isopropylacrylamide) (PNIPAM) is known to exhibit thermosensitive properties according to the theory of the lower critical solution temperature (LCST). PNIPAM has both hydrophilic amide groups and hydrophobic isopropyl groups in its molecule. At low temperatures, there is a strong hydrogen bond between the amide groups and water molecules, causing PNIPAM to swell. When the temperature rises above 32 °C the hydrogen bond between the amide group and the water molecules is broken, the isopropyl group takes over, and PNIPAM shrinks^13^.

The present work presents a combination of solid phase extraction columns whose bed consists of smart molecularly imprinted polymers, and high-performance liquid chromatography as an analytical tool for detecting S-metolachlor herbicide from water samples. This study is subjected to human and environmental risk chemicals occurring in micro quantities, demonstrating the possibility of determining the real concentration of S-metolachlor in the *chemical cocktail* mixture. The composition of molecularly imprinted polymers synthesized according to the principles of *green chemistry*, published in our recent work^14^, combined with the SPE technique, underlines the innovation of the research presented while focusing considerable attention on minimizing environmental risks. The greenification principles used in this work include self-cleaning MIPs, aqueous media as worthy porogen and solvent, generate minimal waste, controlling polymerization without energy waste, eliminate template through use of green solvent, as well as increase operator safety.

## Materials and methods

### Reagents and chemicals

Acrylamide (AA) was purchased from Thermo Scientific Chemicals. Acros Organics provided acrylic acid (AAc). Ammonium persulfate (APS), N-isopropylacrylamide (NIPAM), N,N′-methylenebis (acrylamide) (BIS), and N, N,N′,N′-tetramethylethylenediamine (TEMED) were supplied by Sigma-Aldrich. S-metolachlor (SMCh) was obtained from NOVAGRA. The HPLC grade acetonitrile was purchased from CHEMSOLUTE. Ultrapure water was obtained with a Milli-Q System (Merck Millipore).

### Preparation of the polymers

The synthesis of MIP for S-metolachlor has been published in our previous work^13^. In brief, NIPAM (3.52 mmol), AA (4.64 mmol), and AAc (0.32 mmol) were placed in the vessel as functional monomers, BIS (2.34 mmol) as a cross-liner, and 10 mL of water solution of S-metolachlor (0.27 mmol) as a template molecule. Once dissolved, the mixture was purged with nitrogen for 1 min, and 1 mL of an aqueous solution of APS (0.13 mmol) and TEMED (0.10 mmol) was added to the polymerization mixture and blown with nitrogen for 2 min again. The polymerization reaction was carried out for 24 h on a shaker at room temperature. The bulk polymers were extracted for 24 h in Soxhlet using ethanol and dried at 60 °C. Non-imprinted polymers (NIPs) were synthesized similarly but without template molecules.

### Sorption process

The adsorption capacity *q* (mg × g^−1^), calculated according to the following Eq. (1), is defined as the amount of S-Metolachlor adsorbed at the equilibrium^15^.

1 $$q=\frac{\left({C}_{i}-{C}_{eq}\right)\times V }{m}$$

where ${C}_{i}$ (mg × L^−1^)—initial concentration of S-metolachlor, ${C}_{eq}$ (mg × L^−1^)—equilibrium concentration of S-metolachlor, $V$ (L)—volume of the solution, $m$ (g)—mass of the polymer.

### Regeneration of sorbents

The HPLC apparatus determined the concentration of eluted herbicide. The regeneration factor (RF) was calculated following Eq. (2).

2 $$RF= \frac{{M}_{e}}{{M}_{a}} \times 100\%$$

where ${M}_{e}$ (mg)—mass of eluted S-metolachlor, ${M}_{a}$ (mg)– mass of absorbed S-metolachlor.

### Porosity of sorbents

Pore volume and surface area were obtained by examining nitrogen adsorption at the liquid nitrogen temperature using Micromeritics ASAP 2020 Plus version 2.0 analyzer. Prior to analysis samples (about 0.5 g) were degassed at 40 °C for 24 h. Dose of nitrogen was set to 3 cm^3^ g^−1^, free space of the analyzing tube was measured before performing the analysis. Resultant data were subjected to Braunauer–Emmet–Teller (BET) treatment.

### High-performance liquid chromatography

Analyses were performed on the high-performance liquid chromatograph Shimadzu SCL-40 equipped with an autosampler (model SIL-40), PDA pump, and an ARION^®^ Biphenyl chromatographic column (3 μm, 150 × 4.6 mm). The analysis was performed in isocratic elution using a mixture of water and acetonitrile (1:1, v: v) as a mobile phase. The temperature was kept to 30 °C, the injection volume was set at 30 µL, and the flow rate was maintained at 1 mL × min^−1^. The wavelength has been fixed at 254 nm.

## Result and discussion

### Kinetics of adsorption

**Fig. 1:**
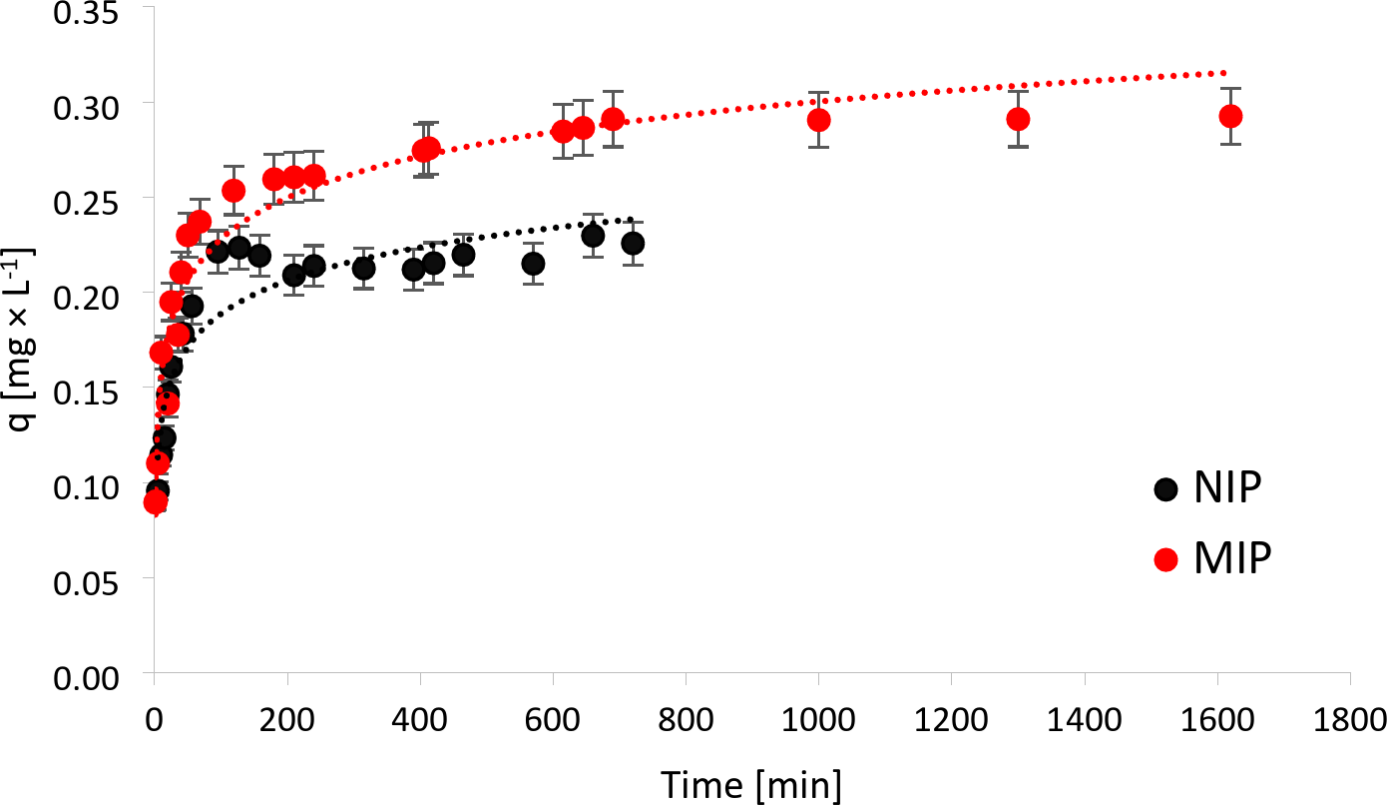
Sorption kinetics for NIP and MIP.

The characterization of the physicochemical properties of the polymers, such as scanning electron microscopy and Fourier transform infrared spectroscopy, was described in our a previous work^14^. In brief, the spectrum indicates the presence of an N–H bending bond at 1650 cm^−1^ and 3470 cm^−1^, as well as a C–N stretching bond at 1040 cm^−1^, which are characteristic of the monomers. Both spectra exhibited a peak at 1715 cm^−1^, 2360 cm^−1^, and 1140 cm^−1^, which indicated the C–O stretching bond of the amide group and C–O–C stretching bond from the cross-linker, respectively. The CH_3_ bending bond was identified at a wavelength of 1460 cm^−1^. At 2850 cm^−1^ as well as 1920 cm^−1^, C–H bonds are observed, while at 3550–3800 cm^−1^, O–H stretching bonds are present. Additionally, the supplementary material contains SEM images. To completely characterize the sorption properties of these polymers sorption kinetic studies were conducted. The adsorption kinetics was investigated with 250 mL of S-metolachlor concentration of 30 mg × L^−1^ and sorbent mass of 2.5 g at room temperature. The concentration of samples taken at different time intervals was determined using the HPLC apparatus. The experimental data obtained from the sorption kinetics process are presented in Fig. 1.

To investigate the mechanism controlling sorption, the collected data were fitted to several kinetic models. *Fick’s second law* models were first adapted to the experimental data to determine whether film diffusion or particle diffusion was the controlling process (Table 1). Equation (3) considers film diffusion as the rate-limiting step^16^. The sorption rate constant ${k}_{a}$ was determined from a plot of $-ln(1-\frac{{q}_{t}}{{q}_{eq}})$ versus time.

3 $${k}_{a}t= -ln \left(1-\frac{{q}_{t}}{{q}_{eq}} \right)$$

where ${k}_{a}$ (1 × min^−1^)—the pseudo-first-order rate constant, ${q}_{t}$ and ${q}_{eq}$ (mg × g^−1^)—the amount of adsorbate adsorbed at time $t$ and at the equilibrium time, respectively.

Approximate expression for particle diffusion-controlled adsorption is given by Eq. (4)^16^. The sorption rate constant ${k}_{b}$ was calculated from a plot of $-\text{l}\text{n}(1-{\left(\frac{{q}_{t}}{{q}_{eq}}\right)}^{2})$ versus time.

4 $${k}_{b}t= -\text{l}\text{n} \left(1-{\left(\frac{{q}_{t}}{{q}_{eq}}\right)}^{2}\right)$$

where ${k}_{b}$ (1 × min^−1^)—the pseudo-second-order rate constant, ${q}_{t}$ and ${q}_{eq}$(mg × g^−1^)—the amount of adsorbate adsorbed at time $t$ and at the equilibrium time, respectively.

**Table 1:** Parameters obtained from the calculation of the sorption kinetics according to *Fick’s law*. Significant values are in bold.

	Film diffusion		Particle diffusion	
	${k}_{a}$ (1 × min^−1^)	*R* ^2^	${k}_{b}$ (1 × min^−1^)	*R* ^2^
NIP	0.032	**0.979**	0.028	0.967
MIP	0.002	0.276	0.012	**0.905**

Analysis of the R^2^ coefficients indicates which type of diffusion is in control of the process. For NIP, film diffusion is the dominant process, but the difference between the R^2^ ratios for the two types of diffusion is negligible. However, for MIP there is a significant difference between the values of the R^2^ coefficient, and particle diffusion controls the process more. A comparison of the data of the rate constants ${k}_{a}$ and ${k}_{b}$ is used to determine which process is faster. These values are very close for NIP, but the particle diffusion process occurs more quickly for MIP.

The limiting stage of the S-metolachlor adsorption process was also estimated using the Weber-Morris model of intraparticle diffusion (Eq. 5; Table 2), which assumes that in many adsorption circumstances, the solute uptake is approximately proportional to t^0.5^ rather than to the contact time. The interparticle diffusion rate constant was calculated from a plot of q_t_ versus *t*^*0.5*^, which should be a straight line if the intraparticle diffusion is the rate-limiting step^17^

5 $${q}_{t}={k}_{WM}{t}^{0.5}+B$$

where ${k}_{WM}$(mg × g^−1^ × min^0.5^)—interparticle diffusion rate constant, ${q}_{t}$ (mg × g^−1^)—the amount of adsorbate adsorbed at time $t$, *B* (mg × g^−1^)—boundary layer thickness.

**Table 2:** Parameters from the Weber-Morris model calculation. Significant values are in bold.

	NIP	MIP
${k}_{WM1}$ (mg × g^−1^ × min^0.5^)	0.017	0.018
${k}_{WM2}$ (mg × g^−1^ × min^0.5^)	0.038	0.063
${k}_{WM3}$ (mg × g^−1^ × min^0.5^)	**0.014**	**0.011**

**Fig. 2:**
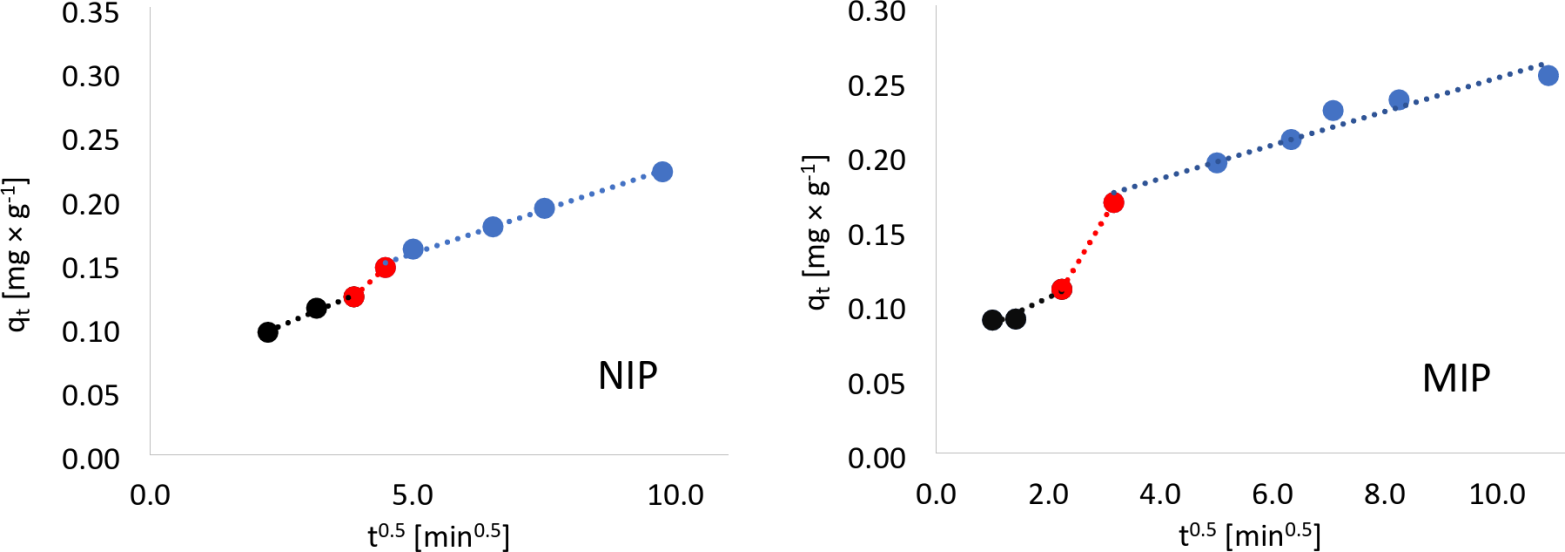
Interparticle diffusion model of S-metolachlor adsorption on NIP and MIP.

However, when analyzing the plots shown in Fig. 2, it is noticeable that there are broken lines rather than straight lines. This is attributable to the fact that various processes are involved in the adsorption process, and not just intraparticle diffusion. Furthermore, none of the curves passes through the origin of the coordinate system, which also confirms that intramolecular diffusion is not the only controlling step. Adsorption on the outer surface of the adsorbent grain, or the immediate adsorption phase, is represented by the first steep section. The second section is equivalent to a gradual, gentle adsorption step in which the process that controls the rate of the overall adsorption process is intraparticle diffusion^18^.

The collected data were fitted to commonly used pseudo-first and pseudo-second-order kinetic models to better understand the mechanisms of sorption kinetics (Table 3). The pseudo-first-order model relies on the assumption that the sorption rate is directly proportional to the difference between the saturation concentration and the amount of solid uptake with time^19^. The linear form of the pseudo-first-order model is presented in following Eq. (6). From the $log\left({q}_{eq}-{q}_{t}\right)$ versus time plot, the rate constant ${k}_{1}$ was calculated^20^.

6 $$\text{log}\left({q}_{eq}-{q}_{t}\right)=\text{log}\left({q}_{eq}\right)-\frac{{k}_{1}t}{2.303}$$

where ${k}_{1}$ (1 × min^−1^)—sorption rate constant, ${q}_{eq}$ and ${q}_{t}$ (mg × g^−1^)—the amount of adsorbate adsorbed at equilibrium time and at time $t$, respectively.

In the pseudo-second-order model, the process is under the control of the chemical sorption reaction at the liquid-solid interface in the adsorbent and predicts the behavior over the entire adsorption range. The linear form of the pseudo-second-order model is described in terms of Eq. (7). The rate constant ${k}_{2}$ was determined from the plot of $\frac{t}{{q}_{t}}$ versus time^20^.

7 $$\frac{t}{{q}_{t}}=\frac{1}{{k}_{2}{q}_{eq}^{2}}+\frac{1}{{q}_{eq}}t$$

where: ${k}_{2}$ (g × (mg × min)^−1^)—sorption rate constant, ${q}_{eq}$ and ${q}_{t}$ (mg × g^−1^)—the amount of adsorbate adsorbed at equilibrium time and at time $t$, respectively.

**Table 3:** Parameters determined by calculating the sorption kinetics according to the pseudo-first and pseudo-second-order models. Significant values are in bold.

	Pseudo-first-order model			Pseudo-second-order model		
	${k}_{1}$ (1 × min^−1^)	*R* ^2^	q_max1_ (mq × g^−1^)	${k}_{2}$ (g × (mg × min)^−1^)	*R* ^2^	q_max2_ (mq × g^−1^)
NIP	0.032	0.979	0.016	0.413	**0.992**	0.226
MIP	0.004	0.874	0.017	0.458	**0.981**	0.263

The absorption kinetics of S-metolachlor for both MIP and NIP can be described by a pseudo-second-order model based on higher values of the R^2^ coefficients. Furthermore, the *q*_*max*_ values calculated according to the pseudo-second-order model are significantly closer to the sorption values from the performed experiment. The sorption rate constant ${k}_{2}$ is higher for the pseudo-second-order kinetics model than the rate constant ${k}_{1}$ for the pseudo-first-order model.

### Optimization of the sorption conditions on SPE columns

Our previous work^14^ described two types of molecularly imprinted polymers that exhibited outstanding sorption properties. Therefore, these materials were chosen for further studies. An investigation into the determination of S-metolachlor concentration using the MIP-SPE technique began with optimizing sorption conditions.

#### Sorption on the aligned vessel column

Initially, the sorption process was carried out on an aligned vessel column. About 0.13 ± 0.01 g of sorbent (MIP and NIP) was placed in a column equipped with wadding and the bed was activated by water. Then 30 mg × L^−1^ S-metolachlor aqueous solution was passed through the column until the bed was penetrated. After filtration through an Acrodisc 0.45 μm syringe filter, the concentration of S-metolachlor was measured by HPLC. The desorption process was carried out with water as an eluent solvent to determine the degree of bed regeneration. The sorption–desorption process on the same materials was repeated three times; the results are presented in Table 4.

**Table 4:** The results of the sorption–desorption process carried out on an aligned vessel column.

	MIP	NIP
Sorption I (mg × g^−1^)	0.71 ± 0.11	0.63 ± 0.05
Desorption I (mg × g^−1^)	0.71 ± 0.09	0.52 ± 0.07
RF (%)	100 ± 2	82 ± 4
Sorption II (mg × g^−1^)	1.33 ± 0.13	1.01 ± 0.08
Desorption II (mg × g^−1^)	1.18 ± 0.06	0.95 ± 0.04
RF (%)	89 ± 4	94 ± 3
Sorption III (mg × g^−1^)	1.31 ± 0.09	0.93 ± 0.07
Desorption III (mg × g^−1^)	1.23 ± 0.08	0.83 ± 0.05
RF (%)	94 ± 6	90 ± 2

To break through the bed of the column, 30 mL of S-metolachlor aqueous solution had to pass through the column for MIP sorbent and 20 mL for NIP sorbent. The desorption process was carried out until a minimum of 80% regeneration of the bed was achieved, with the eluent consumption ranging from 10 to 17 mL. The MIP-SPE column has a higher column capacity, possibly due to the specific binding sites resulting from the formation of imprints on the polymer matrix. This allows it to meet the measurement requirements of large-volume samples. However, this method also has disadvantages, including a long time for the solution and eluent to pass through the bed, driven only by the pressure generated by the liquid in the column of equalizing vessels. Moreover, frequent clogging of the bed and the consumption of large amounts of solvents contributed to the search for improved solutions.

#### Sorption on SPE column with PE frits

A 3 mL SPE column with a PE frit was loaded with approximately 0.09 ± 0.01 g of sorbent (MIP or NIP) and covered with a further PE frit. After activation of the MIP-SPE column with water, 30 mg × L^−1^ S-metolachlor aqueous solution was passed through the column until the bed was punctured and the concentration of the herbicide solution was determined over time using HPLC. To determine the degree of regeneration of the bed, the desorption process was carried out with water as the eluent. The sorption-desorption process was performed twice with the same materials. The data obtained are presented in Table 5.

**Table 5:** The results of the sorption–desorption process conducted on PE fritted SPE columns.

	MIP	NIP
Sorption I (mg × g^−1^)	1.15 ± 0.07	0.96 ± 0.02
Desorption I (mg × g^−1^)	1.04 ± 0.02	0.93 ± 0.01
RF (%)	91 ± 7	97 ± 3
Sorption II (mg × g^−1^)	0.98 ± 0.01	0.85 ± 0.03
Desorption II (mg × g^−1^)	0.97 ± 0.01	0.77 ± 0.04
RF (%)	99 ± 2	90 ± 2

The presence of PE frits in the SPE column prevented the solution from flowing freely through the bed, forcing the use of a vacuum to push the liquid through the column, significantly reducing the sorption and desorption time in the SPE columns compared to the previous system used. The MIP sorption results achieved are slightly lower than the previously discussed sorption on a column of aligned vessels; however, a smaller volume of S-metolachlor solution was required to penetrate the bed. Compared to the first described method, the volume of S-metolachlor solution was reduced from 30 to 18 mL in the first sorption process and to 12 mL in the second sorption process. The first NIP sorption was carried out using the same volume of S-metolachlor solution as in the case of sorption on the column of equalizing vessels, while in the second case, the volume was reduced to 12 mL. The desorption process, in turn, took place with a significant increase in the volume of the eluent − 34 mL for NIP and 31 mL for MIP in the first stage, and 16 and 15 mL for NIP and MIP respectively in the second stage. Despite the shorter analysis time and the high degree of bed regeneration, the sorption-desorption method on SPE columns with PE frits turned out to be unsatisfactory because the column bed consists of tiny pieces of the block polymer that, under the pressure generated by the vacuum, penetrate the pores of the frits, clogging them and blocking further penetration.

#### Sorption on SPE column with wadding and filter paper

To eliminate the effect of PE frits clogging, a layer of two filter papers, wadding, and another two filter papers were tested as a substitute for the standard frits. Approximately 0.08 ± 0.02 g of sorbent (MIP or NIP) was placed in a 3 mL SPE column equipped with a substitute for standard frit and the bed was activated with water. Then, 2 mL of S-metolachlor aqueous solution was dosed three times onto the column, regulating the liquid flow using the under pressure generated by the vacuum pump. The herbicide concentration was determined by high-performance liquid chromatography. The desorption process was carried out to determine the degree of bed regeneration. For this purpose, three portions of water, constituting the eluent, with a volume of 2 mL were passed through the column. The results of this are summarized in Table 6.

**Table 6:** The results of the sorption–desorption process conducted on SPE columns with wadding.

	MIP	NIP
Sorption (mg × g^−1^)	0.91 ± 0.28	0.52 ± 0.30
Desorption (mg × g^−1^)	0.88 ± 0.35	0.56 ± 0.25
RF (%)	98 ± 2	86 ± 1

The use of wadding and filter paper had the desired effect, enabling the sorption-desorption process to take place without clogging. Moreover, the use of just 6 mL of the S-metolachlor solution resulted in MIP sorption values almost as high as those using 30 mL of the solution for sorption carried out on an aligned vessel column. Furthermore, 6 mL of eluent was sufficient to regenerate almost 100% of the MIP bed and almost 90% of the NIP bed. The almost twofold difference between MIP and NIP sorption is evidence of the specific binding of template molecules by the molecularly imprinted polymer.

Due to the significantly reduced amount of solvents, in line with the *green chemistry* strategy, the shortened analysis time, and the evidence for the presence of particular cavities on the structure of MIP capable of binding of S-metolachlor molecules, the SPE column method using two layers of filter paper, cotton wool, and subsequent layers of filter paper was chosen as the most suitable method for carrying out sorption-desorption processes of S-metolachlor.

#### Desorption on SPE column in various temperatures

After optimizing the sorption and desorption conditions on SPE columns, the desorption process was carried out at various temperatures to confirm the thermosensitive properties of PNIPAM. The desorption of adsorbed S-metolachlor molecules was tested at three different temperatures: 4 °C, 22 °C (room temperature) and 50 °C for 75 min. The desorption process was carried out using 1 mL of water as eluent. The results of the research are presented in Table 7.

**Table 7:** The values of the desorption process conducted in various temperatures.

	MIP	NIP
RF 4 °C (%)	66 ± 12	92 ± 11
RF 22 °C (%)	55 ± 3	67 ± 2
RF 50 °C (%)	46 ± 9	76 ± 8

The above results confirm the change in the physicochemical properties of PNIPAM in response to a change in temperature. At lower temperatures, the polymer chain swells and becomes more flexible, due to the strong hydrogen bonds between the amide groups and water molecules. The release of adsorbed S-metolachlor molecules from the polymer structure is facilitated by the relaxation of the network and change of the polymer chain’s conformation. Therefore, the temperature of 4 °C was selected as the optimal desorption temperature, in line with the LCST theory. At low temperatures, there is a strong hydrogen bond between the amide groups and water molecules, causing PNIPAM to swell. When the temperature rises above 32 °C the hydrogen bond between the amide group and water is broken, the isopropyl group takes over and PNIPAM shrinks.

#### Regeneration of the column bed

After optimizing the sorption and desorption conditions on the SPE columns, it was investigated how many cycles the column bed could be reused while maintaining the initial sorption parameters. Approximately 0.05 ± 0.01 g sorbent (MIP or NIP) was weighed into 3 mL SPE columns and the bed was activated by wetting with water. In the next step, 2 mL of S-metolachlor aqueous solution (C_e_ = 10 mg × L^−1^) was added to the column and incubated for 3 h. In the next step, the adsorbed S-metolachlor particles were desorbed at 4 °C using water as eluent until at least 93% of the deposit was regenerated. The results are summarized in Table 8.

**Table 8:** The results of sorption-desorption process cycles performed on the MIP sorbent.

	MIP	NIP
Sorption I (mg × g^−1^)	0.50 ± 0.02	0.35 ± 0.06
RF (%)	93 ± 0	99 ± 1
Sorption II (mg × g^−1^)	0.51 ± 0.01	0.42 ± 0.02
RF (%)	98 ± 3	99 ± 1
Sorption III (mg × g^−1^)	0.53 ± 0.04	0.42 ± 0.03
RF (%)	98 ± 1	98 ± 1
Sorption IV (mg × g^−1^)	0.58 ± 0.17	0.43 ± 0.06
RF (%)	77 ± 1	100 ± 0

The column bed has been tested to be used as many as 3 times before it loses its sorption properties. By the fourth sorption–desorption cycle performed, the bed consist of MIP sorbent was blocked, preventing desorption above 80%.

### Binding site selectivity

Once the conditions were optimized, the method was used for a selectivity analysis to assess the differences in the binding of the analyte between the MIP and NIP sorbents. Approximately 0.08 ± 0.02 g of sorbent (MIP or NIP) was introduced into a 3 mL SPE column and activated with water. The column was then treated with three 2 mL portions of a aqueous solution containing a mixture of plant protection products: atrazine (concentration 0.05 mmol × L^−1^), S-metolachlor, glyphosate, and fenoxaprop-P-ethyl (concentration 0.1 mmol × L^−1^ for each), regulating the liquid flow using the underpressure generated by the vacuum pump. The concentration of the solution was determined by HPLC, and the chromatograms are provided in the supplementary information. The results are presented in Table 9.

The recognition selectivity is rated by the distribution coefficient (${K}_{i\left(j\right)}$) demonstrating the difference between the concentration ($i$—S-metolachlor, $j$—other plant protection products) in solution before and after absorption, calculated according to the following Eq. (8)^21^.

8 $${K}_{i\left(j\right)}= \frac{{(C}_{i\left(j\right)}-{C}_{i\left(j\right)eq}) \times \rho }{ {C}_{i\left(j\right)eq}}$$

Where: ${C}_{i\left(j\right)}$ (mg × L^−1^)—initial concentration ($i$—S-metolachlor, $j$—other plant protection products), ${C}_{i\left(j\right)eq}$ (mg × L^−1^)—equilibrium concentration ($i$—S-metolachlor, $j$—other plant protection products) $\rho$(g × L^−1^)—density of the solution.

The imprinting factor ($IF$) is the ratio of the distribution coefficients of imprinted polymer and the non-imprinted polymer was calculated with Eq. (9)^20^

9 $$IF= \frac{{K}_{i}^{MIP}}{{K}_{i}^{NIP}}$$

Where: *K*_*i(j)*_—distribution coefficient ($i$—S-metolachlor, $j$—other plant protection products) of a substance on an imprinted polymer (${K}^{MIP}$) and non-imprinted polymer (${K}^{NIP}$).

The selectivity factor ($S$) was calculated using the following Eq. (10)^21^

10 $$S=\frac{{IF}_{i}}{{IF}_{j}}$$

where: ${IF}_{i}$—imprinting factor of the S-metolachlor, ${IF}_{j}$—imprinting factor of analogue molecules.

**Table 9:** The results of the competitive study.

Parameter	S-Metolachlor	Atrazine	Fenoxaprop-*P*-ethyl	Glyphosate
*K*_*MIP*_	5.94	2.37	1.74	0.57
*K*_*NIP*_	0.61	2.48	2.06	1.93
*IF*	9.7	1.0	0.8	0.3
*S*	–	10.1 ± 0.1	11.5 ± 0.2	33.0 ± 0.4

From the sorption data collected, the distribution coefficients were calculated for each of the compounds. The imprinting factor was used as a measure of MIP recognition characteristics. The *IF* for the synthesized MIP was calculated as 9.7, which is approximately 10 times higher than for atrazine, almost 12 times higher than for fenoxaprop-P-ethyl, and above 30 times higher than for glyphosate. The high selectivity values confirm the specific recognition properties of S-metolachlor by MIP due to specific cavities on the polymer matrix.

### Sorption from real samples

After optimization of the conditions, the method has been applied to the analysis of real samples. The solution was prepared from tap water to which S-metolachlor was added and the pH of the solution was adjusted with HCl to the pH of distilled water. A more detailed description of the conditions for selecting the pH can be found in our previous work^14^, and chromatograms are provided in the supplementary information. Approximately 0.05 ± 0.01 g of the sorbent was loaded into a 3 mL SPE column and activated with water. Then 2 mL portions of a tap water solution of S-metolachlor were passed through the column, adjusting the liquid flow by vacuum, and the concentration of herbicide was indicated using HPLC. The desorption of S-metolachlor was carried out with water as an eluent. The results are given in Table 10.

**Table 10:** Results of the sorption-desorption process carried out on real samples.

	MIP	NIP
Sorption (mg × g^−1^)	0.85 ± 0.08	0.71 ± 0.07
RF (%)	100	100

The results obtained (Table 9) show that there were no significant differences between the real and the experimental data and confirm the validity of the proposed polymer composition for the determination of S-metolachlor from real samples.

### Porous structure analysis of obtained polymers

To complete the physicochemical characterization of the materials obtained which was started in our last paper^13^, the porous structure analysis was performed. This analysis was also supposed to give us an indirect answer to the question that the unique sorption properties of MIP in relation to S-metolachlor were ensured by the imprints produced during polymerization in the polymer matrix. The measurement results with ASAP are presented in Table 11. The porous structure of the analyzed materials was characterized using nitrogen adsorption. The values of the average pore volume and the specific surface area for the selected pair of MIP and NIP were also very similar. According to IUPAC, both polymers studied, characterized by average pore size in a range of 2 and 50 nm, can be classified as mesoporous materials^22^. It can be observed that the porosity value is very small; however, it was determined for a dry sample, and our material is a typical hydrogel with a high water capacity (after swelling, 1 g of NIP weighed 6.14 g, and 1 g of MIP weighed 6.25 g)^14^, where the surface develops in an aqueous environment and provides access to potential sorption sites for S-metolachlor, hence such a relatively high sorption capacity despite the small active surface area.

**Table 11:** Characteristic of the selected pair of polymers.

Sample	BET surface (m^2^ × g^−1^)	Pore volume (cm^3^ × g^−1^)	Average pore size (nm)
NIP	0.15 ± 0.02	4.0 × 10^−5^	3.3
MIP	0.19 ± 0.02	6.0 × 10^−5^	3.3

## Conclusions

The article describes the further characterization of the synthesized smart *green* molecularly imprinted polymers as selective sorbents for S-metolachlor herbicide in a solid phase extraction column. In connection with HPLC, this method can be used for the detection of traces of S-metolachlor in water samples. The extraction procedure was optimized using different columns, the best being an SPE column with a layer of two filter papers and wadding as a substitute for standard PE frits. Molecularly imprinted polymers have a high IF value for metolachlor (IF = 10), while for the other plant protection products the value does not exceed 1. The sorption capacity of the MIP-SPE column was almost twice that of the non-printed polymer column, confirming the specific interactions between the matrix and S-metolachlor. The desorption process was optimized at 4 °C, confirming the thermosensitive properties of NIPAM. MIPs have been tested to be recyclable up to three times, with an average regeneration factor of 98%. The MIP-SPE column is a good method for sample enrichment and preconcentration, characterized by speed, high selectivity, easier analysis, and no requirement for expensive instrumentation.

## Electronic supplementary material

Below is the link to the electronic supplementary material.

Supplementary Material 1

## Data Availability

The data will be available on the request. To obtain research data, please contact Dominika Rapacz; email: dominika.rapacz@pwr.edu.pl.
