# Supplementary material for: Preparation and characterization of SPE column with smart green molecularly imprinted polymers materials for selective determination of S-metolachlor herbicide
Source: Sci Rep. 2025 Jan 24;15:3153. doi: 10.1038/s41598-025-87685-2 (PMC11761453; doi:10.1038/s41598-025-87685-2)

**Preparation and characterization of SPE column with smart green molecularly imprinted polymers materials for selective determination of S-metolachlor herbicide**

Dominika Rapacz^1^,*, Katarzyna Smolińska-Kempisty^1^, Joanna Wolska^1^

^1^ Wroclaw University of Science and Technology, Department of Process Engineering and Technology of Polymer and Carbon Materials, Wybrzeże Wyspiańskiego 27, 50-370 Wrocław, Poland

* Corresponding author. E-mail address: dominika.rapacz@pwr.edu.pl

**Supplementary information**

1. The characterization of the physicochemical properties of the polymers.

Scanning electron microscopy images were taken to visualize the surface morphology of the MIP and NIP polymers.


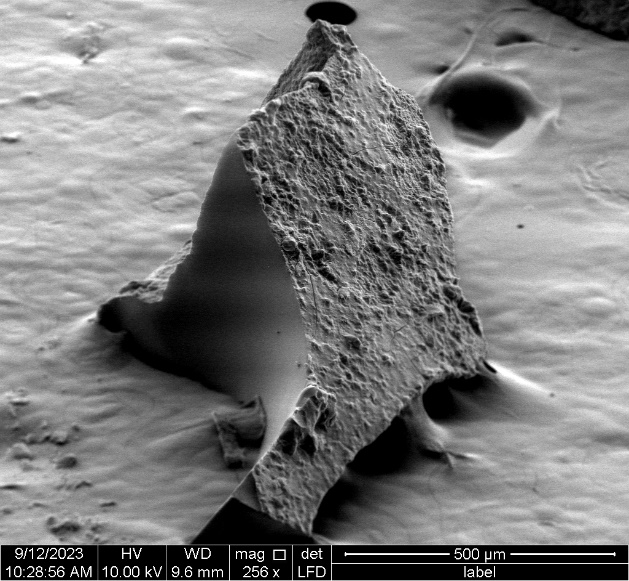

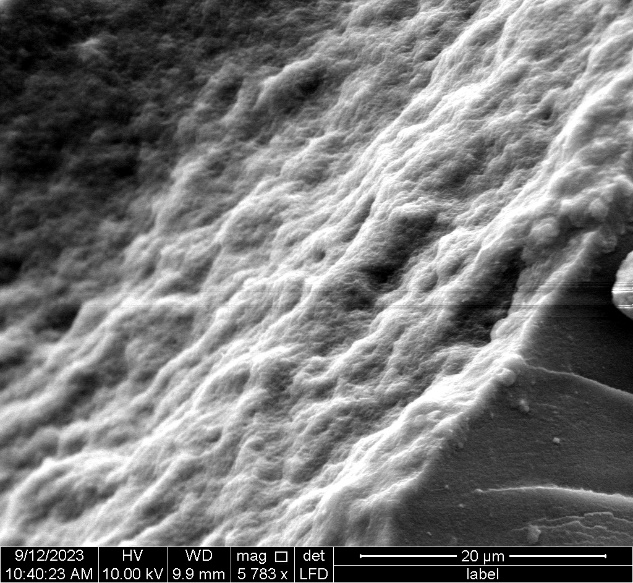


A B


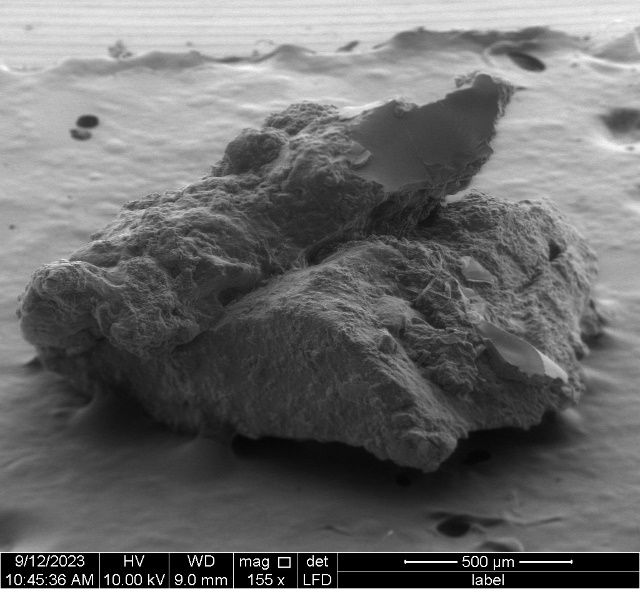

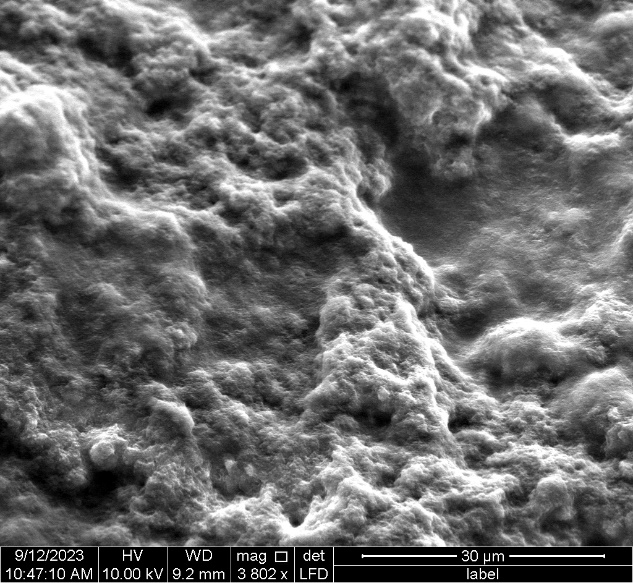


C D

**Figure 1. The SEM images of MIP (A, B) and NIP (C, D).**

1. Chromatograms of analog compound and S-metolachlor mixture solution.

To enhance the quality of the research, the chromatograms obtained from the sorption of S-metolachlor from a mixture of plant protection products are presented below. The mixture comprised the following compounds: glyphosate Rt = 1.7, atrazine Rt = 4.9, phenoxaprop-P-ethyl Rt = 7.0 and S-metolachlor Rt = 13.7.

- 1. Sorption chromatograms on MIPs.


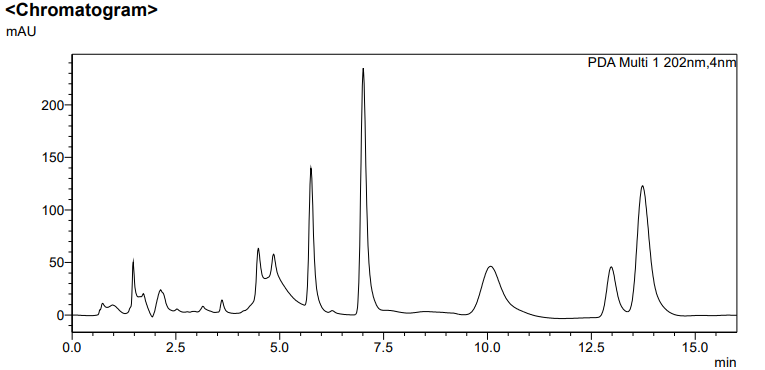


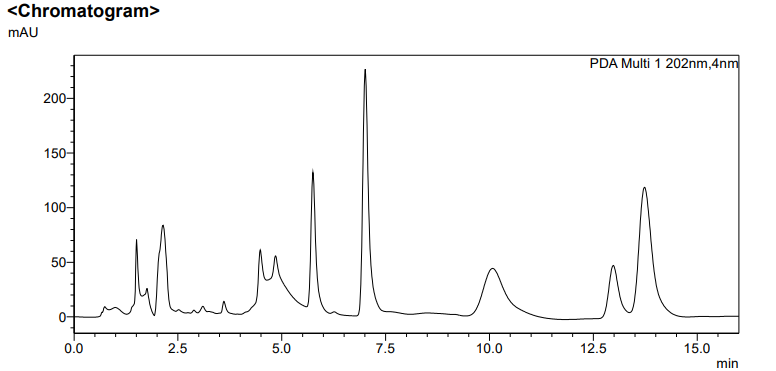


- 1. Sorption chromatograms on NIPs.


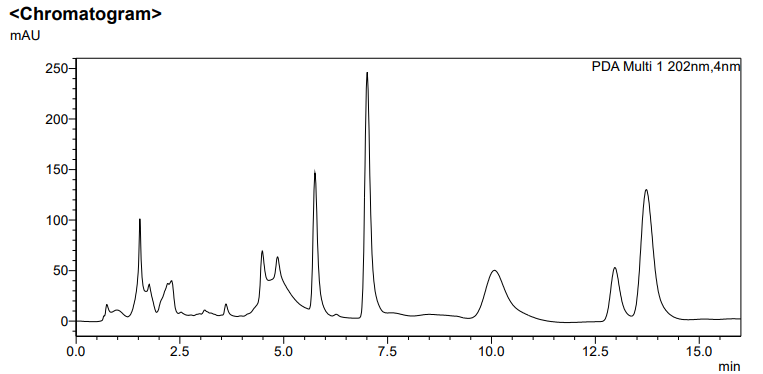


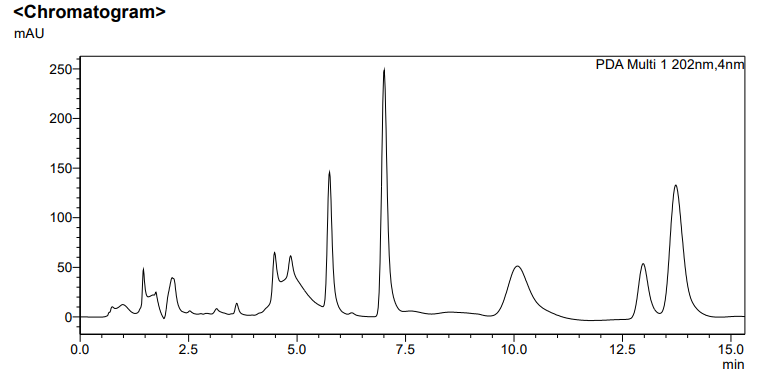


1. Chromatograms related to pH optimization.

In order to determine the solutions of the real samples, it was necessary to optimize the pH, which is described in more detail in article in section 3.4. Below are the chromatograms for the sorption of S-metolachlor from a solution at pH ~ 7 and at pH ~ 5, similar to the pH of distilled water.

- 1. Sorption of S-metolachlor at pH ~7.
     1. MIP


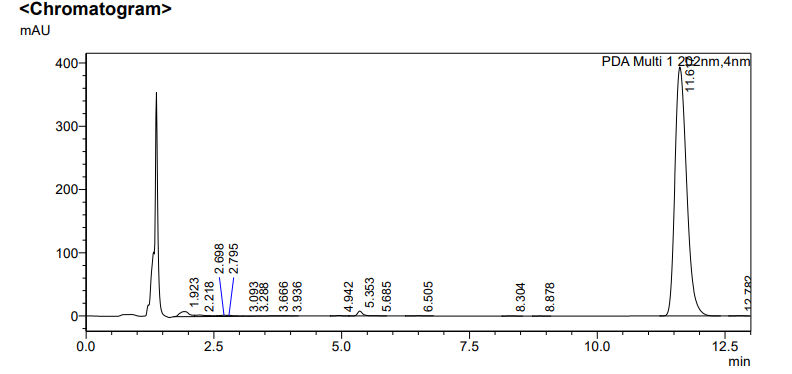


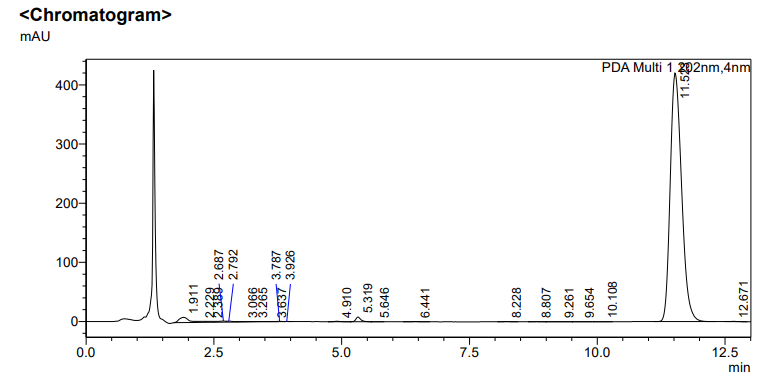


- - 1. NIP


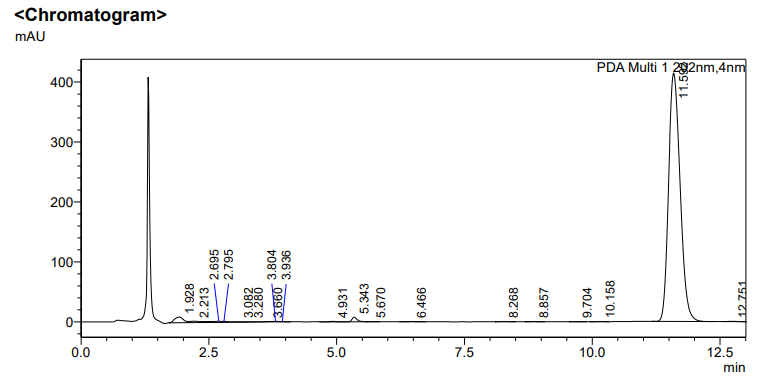


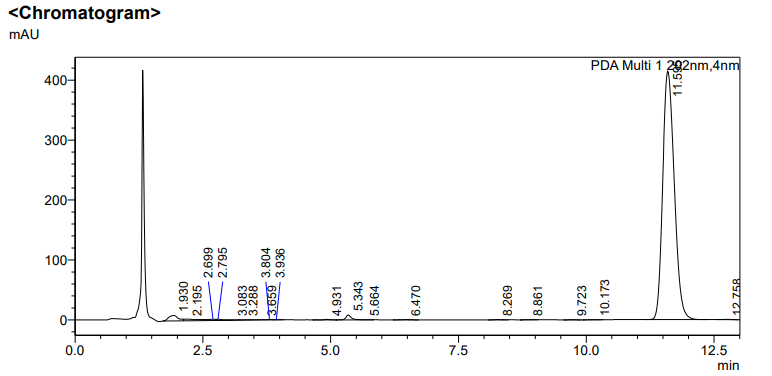


- 1. Sorption of S-metolachlor at pH ~5.
     1. MIP


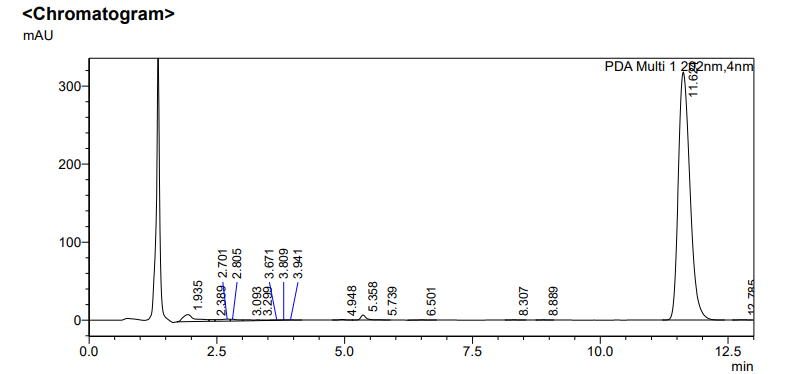


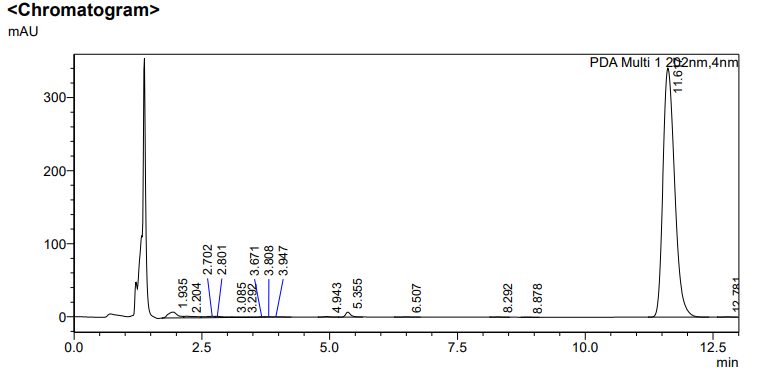


- - 1. NIP


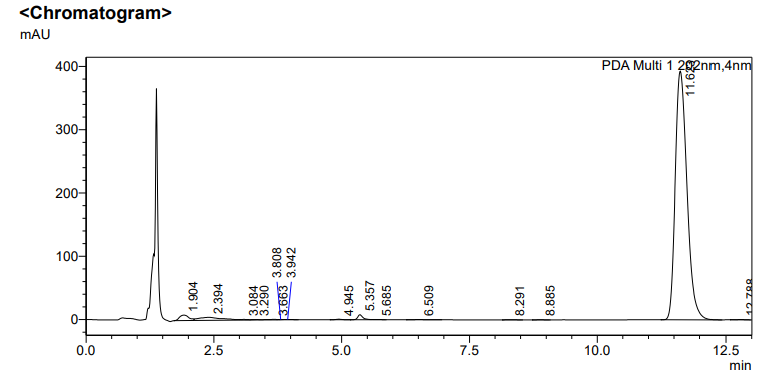


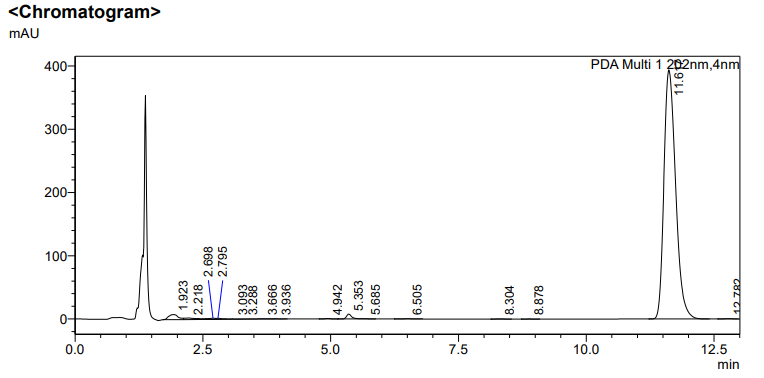

Supplement: Supplementary file 1 — Supplementary Material 1 [file 41598_2025_87685_MOESM1_ESM.docx]
